# Supplementary material for: H3K9me3-mediated epigenetic regulation of senescence in mice predicts outcome of lymphoma patients
Source: Nat Commun. 2020 Jul 20;11:3651. doi: 10.1038/s41467-020-17467-z (PMC7371731; doi:10.1038/s41467-020-17467-z)
Supplement: Supplementary file 1 — Supplementary Information [file 41467_2020_17467_MOESM1_ESM.pdf]

SUPPLEMENTARY INFORMATION

H3K9me3-mediated epigenetic regulation of senescence in mice predicts outcome of lymphoma patients

Schleich et al

Supplementary Figures

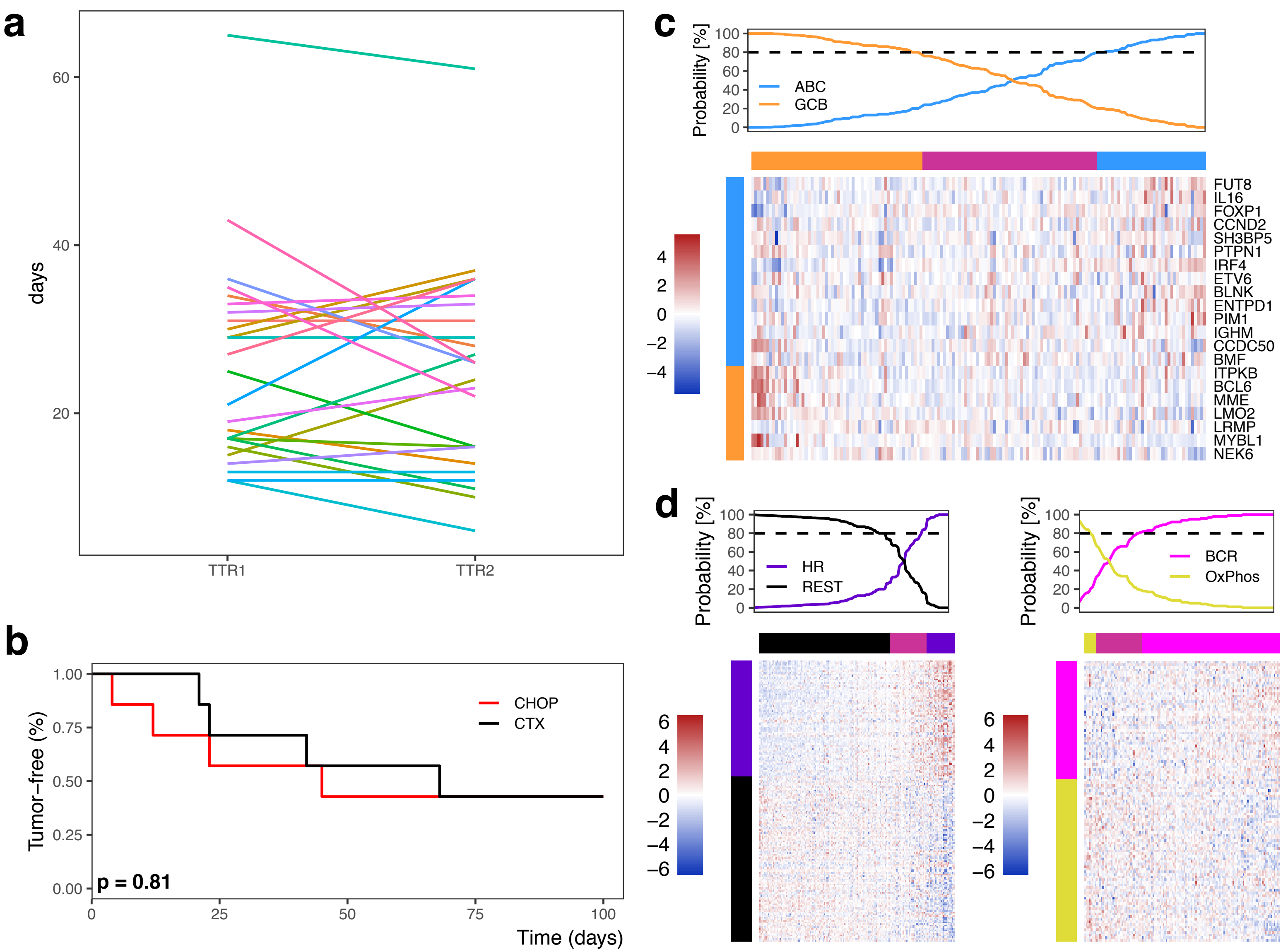

Supplementary Fig. 1 DLBCL- reminiscent features of the Eµ-*myc* model

a TTR1 and TTR2 (connected by a line) for 24 pairs of recipient mice transplanted with the same individual primary RP lymphoma (a comparable degree of concordance applies to matched recipients of individual NR lymphomas (data not shown). **b** Tumor-free survival of mice bearing matched paired primary lymphomas treated with CTX (black line) or CHOP (red line). n = 7 individual lymphomas each. Survival analysis was done using the *survival* package in R. Statistical significance of differences in the survival times were assessed using the log-rank test. **c** Heatmap and associated COO subtype probability for primary Eµ-*myc* lymphomas (n = 154). **d** Heatmaps and associated CCC subtype probabilities for Eµ-*myc* lymphomas as in **c**.

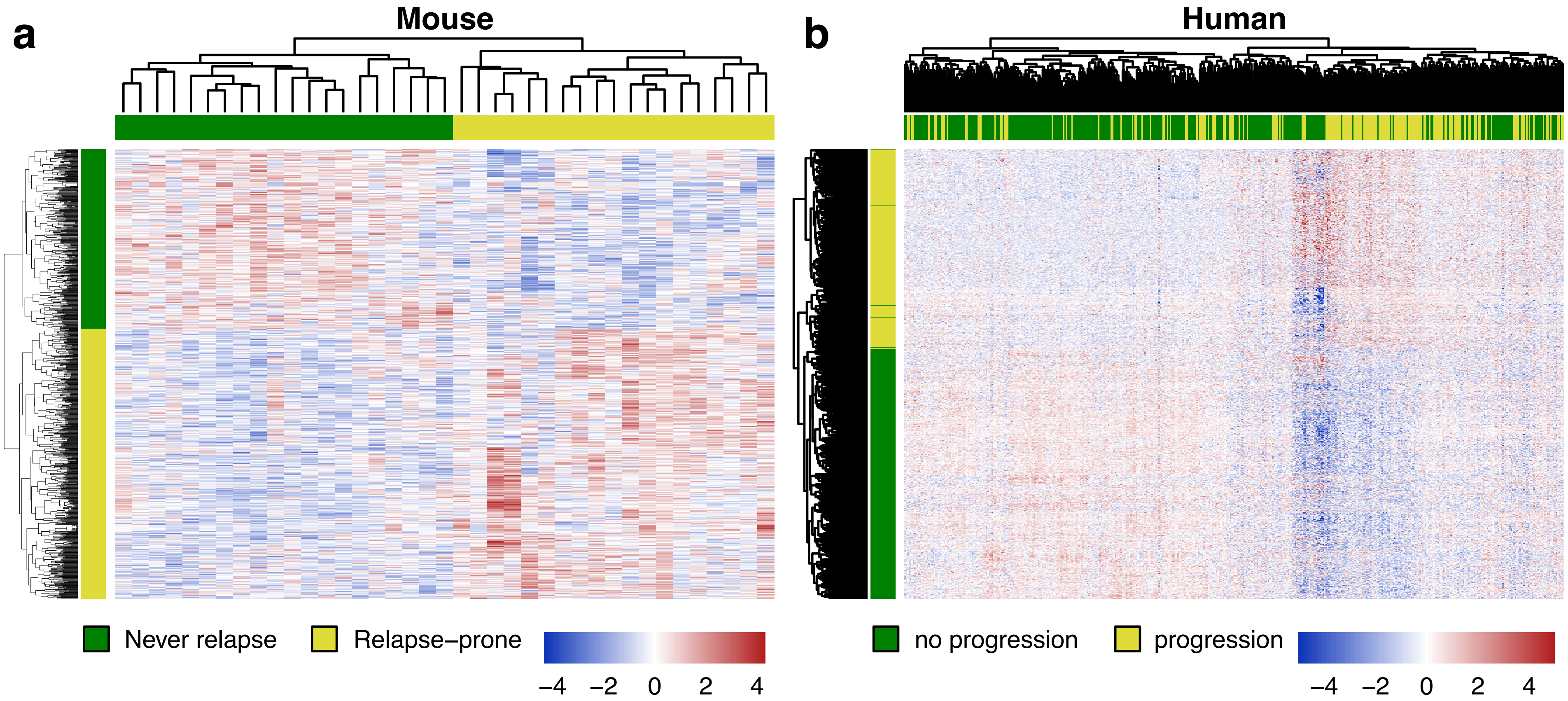

c

	Never relapse	Relapse−prone
**1**	17	0
**2**	3	19

p−value = 2.57e−08

d

	no progression	progression
**1**	212	85
**2**	80	93

p−value = 8.52e−08

Supplementary Fig. 2 Overlapping differential gene expression in progressing *vs.* non-progressing Eμ-*myc* mouse lymphomas and human DLBCL

a Heatmap of the 1,000 most strongly differentially expressed genes in 39 primary Eμ-*myc* lymphomas separating NR from RP samples. **b** Heatmap of the most strongly significantly differentially expressed genes (BH-adjusted p value < 0.05, log-FC > 0) of 470 cured *vs.* relapsing DLBCL patients (GSE31312). Because of little very high and low z-scores, they were limited in the interval [-5,5] after hierarchical clustering for presentation purposes only. **c** Contingency table of the clinical response groups compared to cluster 1 and 2 assignment as shown in Figure 2a (color bars) for Eμ-*myc* lymphomas presented in **a**. **d** As in **c**, but for DLBCL patients (presented in **b**) as shown in Fig. 2b. *P* values in **c** and **d** were computed by Fisher's exact test.

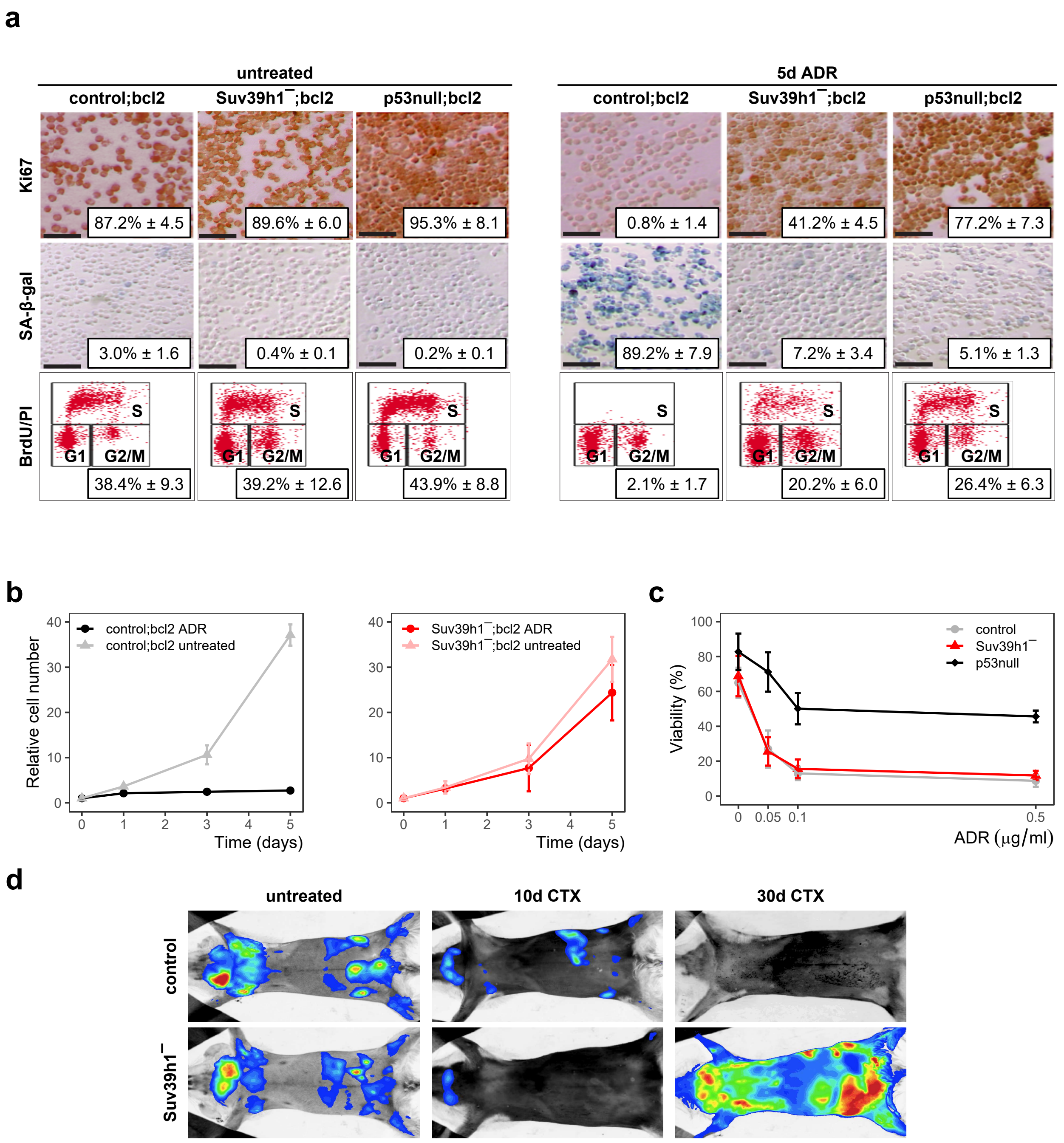

Supplementary Fig. 3 Lack of Suv39h1 selectively impairs senescence in Eμ-*myc* lymphomas but leaves apoptosis intact

a Flow cytometric PI/BrdU-based cell-cycle analysis (quantified is the percentage of cells in S-phase) as well as Ki67 and SA-β-gal stainings in cytospin preparations of Bcl2-protected control, Suv39h1⁻, or p53*null* lymphomas after 5d ADR treatment *vs.* no treatment *in vitro*. Results show mean percentages of cells positive for the respective marker ± s.d. (n = 3 primary lymphomas per genotype). Scale bars represent 50 μm. Gating strategy of the FACS analysis is presented in supplementary figure 9. **b** Growth curve analyses of control;bcl2 and Suv39h1⁻;bcl2 lymphoma cells with or without ADR

treatment as in **a** (n = 3 primary lymphomas per genotype). Error bars represent ± s.d. **c** 24-hour-viability assay of control, Suv39h1^{-}, and p53*null* lymphomas without exogenous Bcl2 overexpression exposed to the indicated ADR doses or left untreated. Results show mean percentages of viable cells ± s.d. (n = 6 primary lymphomas per genotype). **d** Another representative whole-body luciferase imaging set of mice harboring an independent Suv39h1^{-} *vs.* an independent control lymphoma (not bcl2-engineered) before treatment as well as 10 and 30 days after CTX treatment (refers to Fig. 3f).

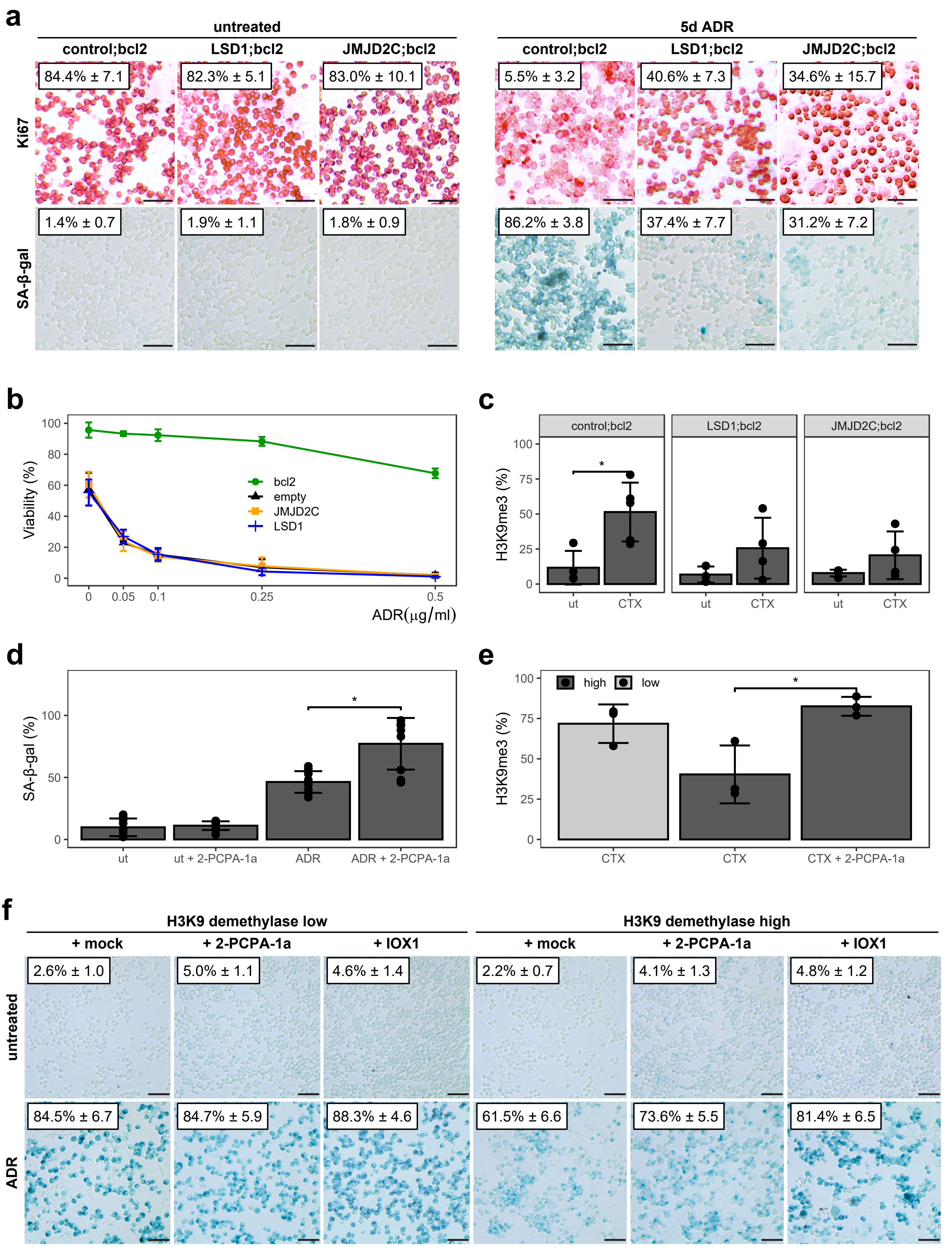

Supplementary Fig. 4 Altered TIS capability in H3K9me3-active demethylase-modulated Eµ-*myc* lymphomas

a Ki67 and SA-β-gal stainings in cytospin preparations of Bcl2-protected control or LSD1- or JMJD2C-overexpressing lymphomas after 5d ADR treatment *vs.* no treatment *in vitro*. Results show mean percentages of cells positive for the respective marker ± s.d. (n = 3 primary lymphomas per genotype). **b** 24-hour-viability assay of lymphomas as in **a**, albeit without exogenous Bcl2 overexpression, compared to Bcl2-protected and empty control lymphoma exposed to the indicated ADR doses or left untreated. Results show mean percentages of viable cells ± s.d. (n = 3 primary lymphomas per genotype). **c** H3K9me3 immunohistochemistry in lymphoma sections after 5-day CTX treatment of mice bearing Bcl2-protected control (n = 4 primary lymphomas for UT, 5 for CTX) or LSD1- (n = 3 primary lymphomas for UT, 4 for CTX) or JMJD2C-overexpressing lymphomas (n = 4 primary lymphomas for both UT and CTX). Results show mean percentages of positive lymphoma cells ± s.d. **d** SA-β-gal activity measured in LSD1;bcl2 lymphomas five days after *in vitro*-exposure to ADR or ADR plus 150 µM of the LSD1 inhibitor 2-PCPA-1a or left untreated. Results show mean percentages of positive cells ± s.d. (n = 3 primary lymphomas). **e** H3K9me3 immunohistochemistry in lymphoma sections of mice bearing lymphomas (without exogenous Bcl2 overexpression) assigned to high *vs.* low (above/below median, n = 3 primary lymphomas for each group) H3K9-active demethylases expression levels (according to a GSEA of their Affymetrix GEP; GO:0032454) and treated as in Fig. 4d. **f** SA-β-gal activity measured in lymphomas stratified by their H3K9 demethylase activity status according to Fig. 4e five days after *in vitro*-exposure to ADR or ADR plus 150 µM of the LSD1 inhibitor 2-PCPA-1a or ADR plus 10 µM of the JMJD2 inhibitor IOX1, or left untreated. Results show mean percentages of positive cells ± s.d. (n = 4 primary lymphomas). "*" in **c** - **e** represents statistically significant difference by unpaired t-test. p = 0.0123 in **c**, p = 0.0985 in **d**, p = 0.0179 in **e**. All scale bars in this figure represent 50 µm.

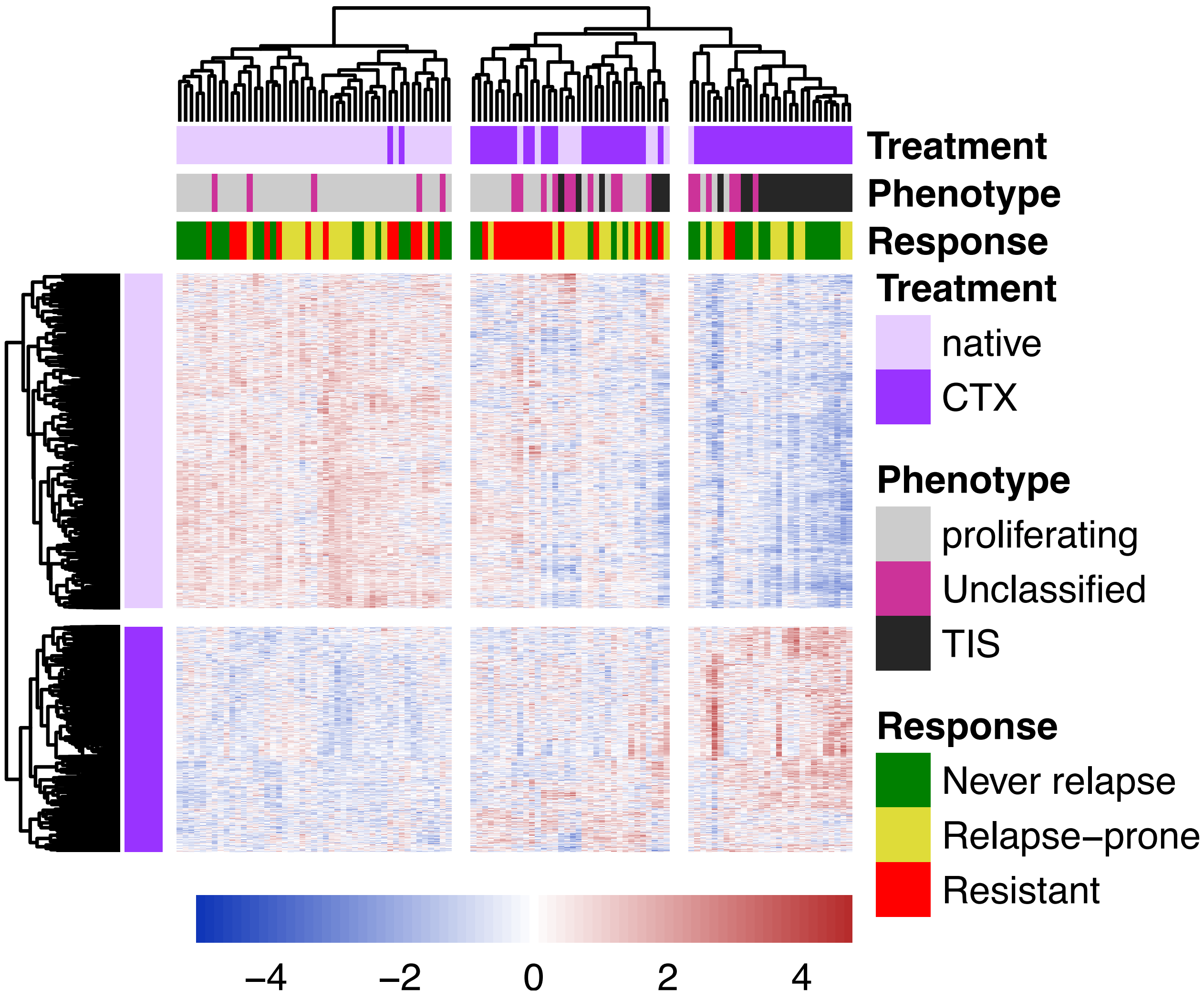

Supplementary Fig. 5 Gene expression clustering among different treatment, TIS capacity and response groups

Heatmap of significantly differentially expressed genes (BH-adjusted p value < 0.05) of 54 4-hour-CTX-challenged compared to 55 untreated lymphoma-bearing mice of the clinical trial-like cohort (labeled according to response groups NR, RP and RES). The phenotype-indicating color bar refers to LPS predictions *(i.e.* TIS capacity) from Fig. 5C. Note that the CTX-challenged lymphomas largely fell into two out of three top-hierarchy clusters, while RES lymphoma samples were found particularly enriched for in a cluster with an expression profile between the mostly untreated samples on one side and the short-term CTX-pulsed and TIS-capable group on the other side, and were largely predicted to have a proliferating non-senescent phenotype.

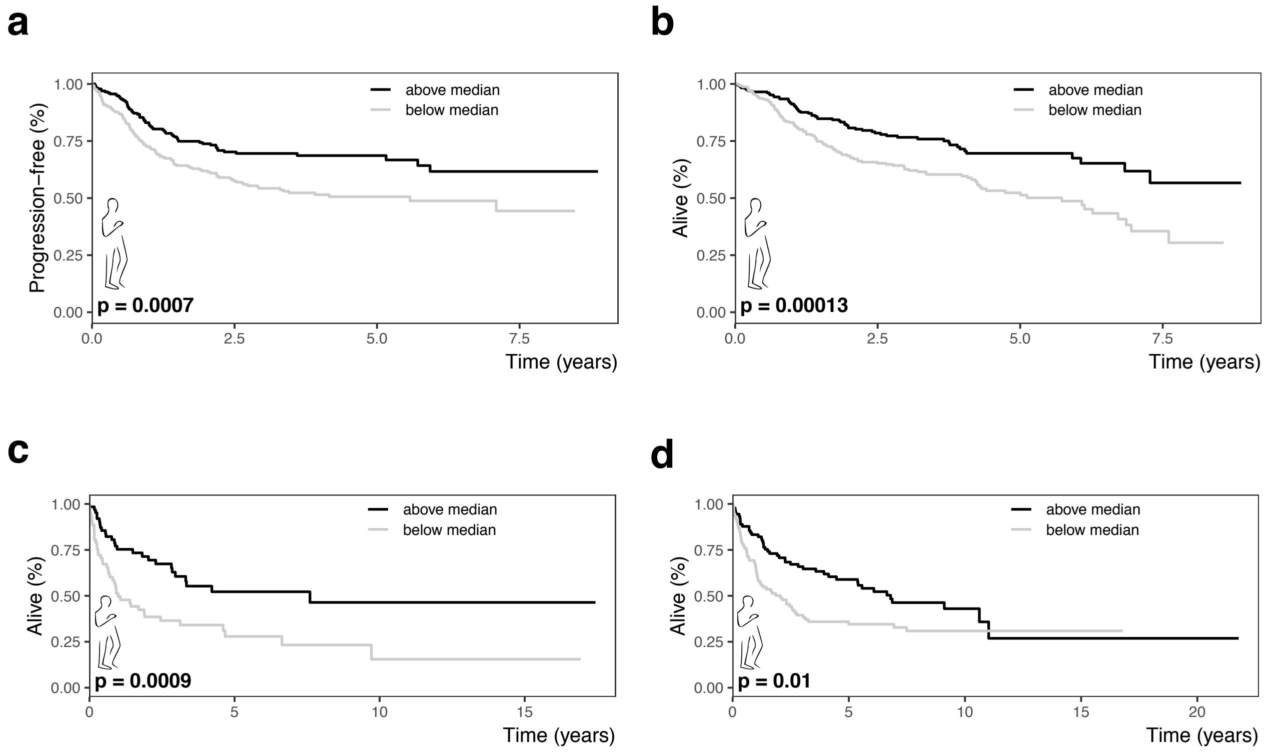

Supplementary Fig. 6 Further validation of the SUVARness gene signature

a PFS in an independent cohort (GSE31312, but stratified as in Fig. 6c, d) of 470 R-CHOP-treated DLBCL patients. Above median: n = 235 (black line), below median: n = 235 (grey line). **b** OS for patients stratified as in **a**. **c** OS in an independent cohort (GSE4475, but stratified as in Fig. 6c, d) of 127 CHOP-treated DLBCL patients. Above median: n = 64 (black line), below median: n = 63 (grey line). **d** As in **c**, but showing another independent CHOP-treated DLBCL cohort (GSE10846) comprising 181 patients. Above median: n = 90 (black line), below median: n = 91 (grey line). Survival analysis was done using the *survival* package in R. Statistical significance of differences in the survival times were assessed using the log-rank test.

Supplementary Fig. 7 Original scans of gels in Figure 3d

Control

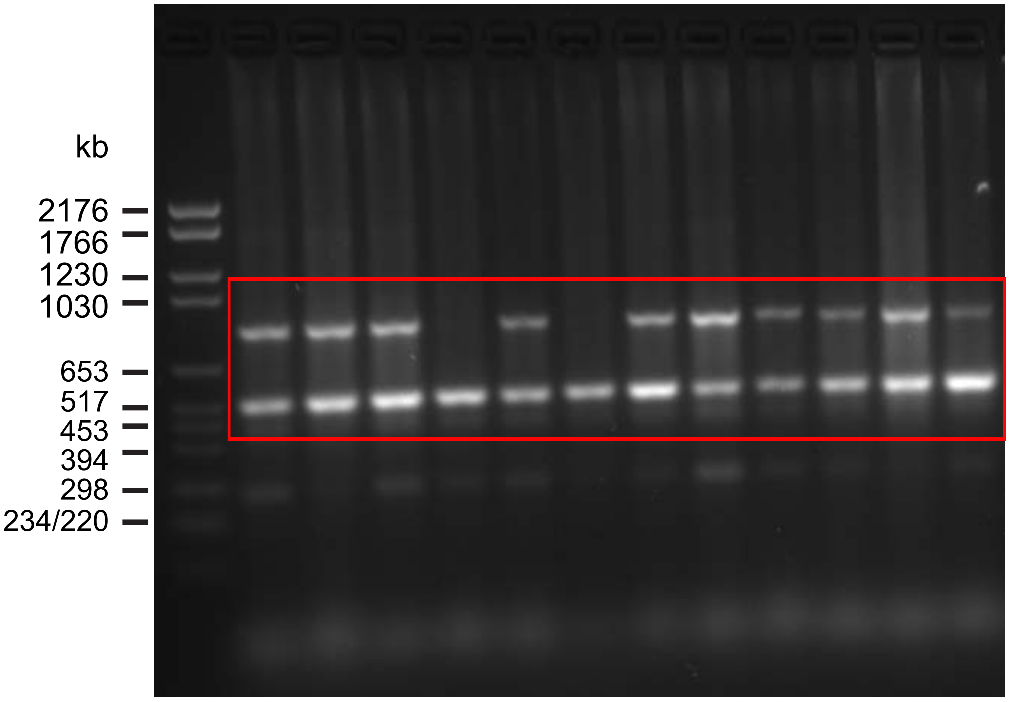

Suv39h1⁻

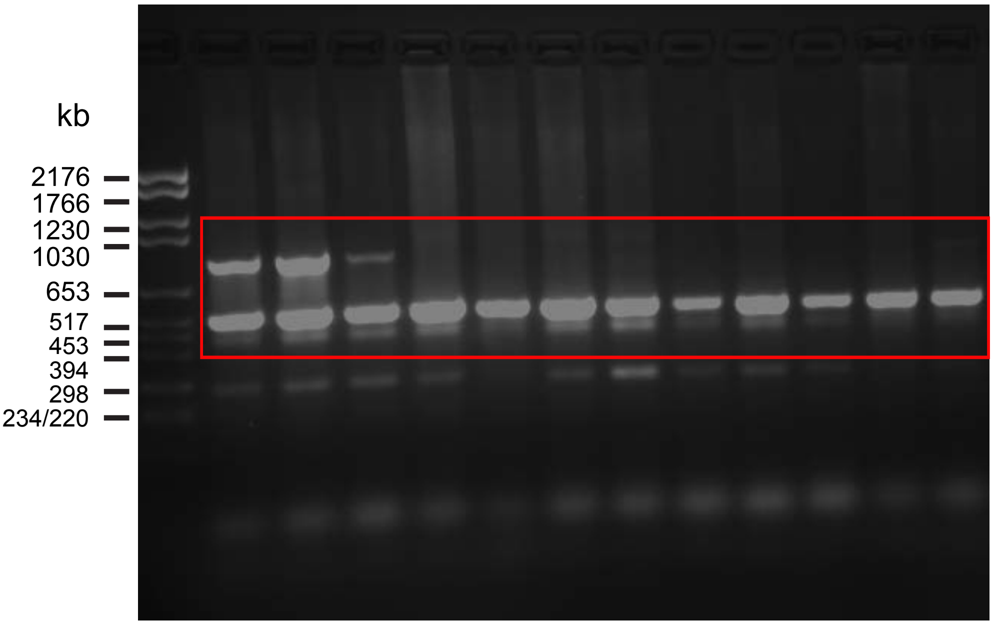

Supplementary Fig. 8 Original scans of immunoblots in Figure 4a

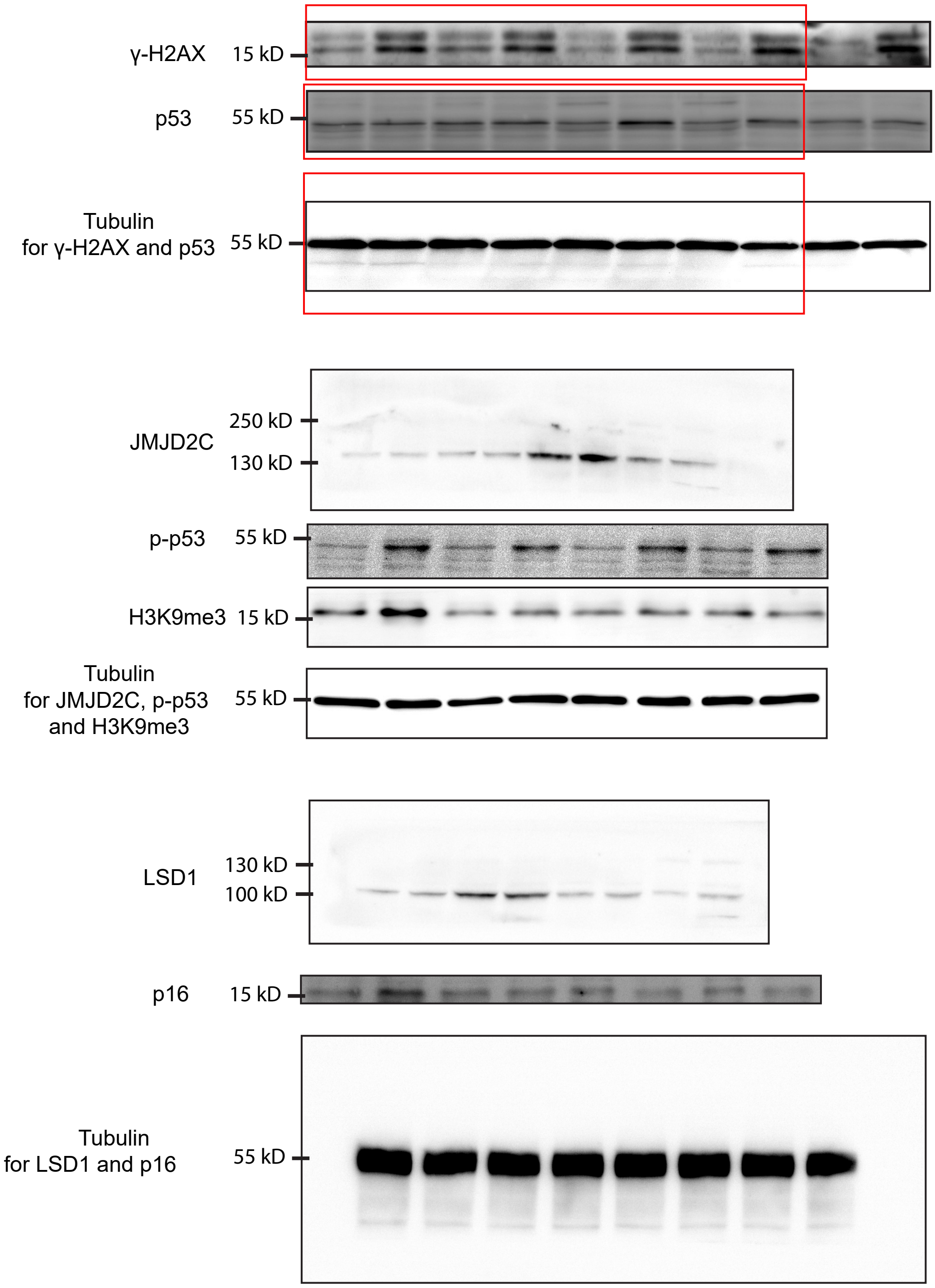

Supplementary Fig. 9 Gating strategies used for supplementary Figure 3a

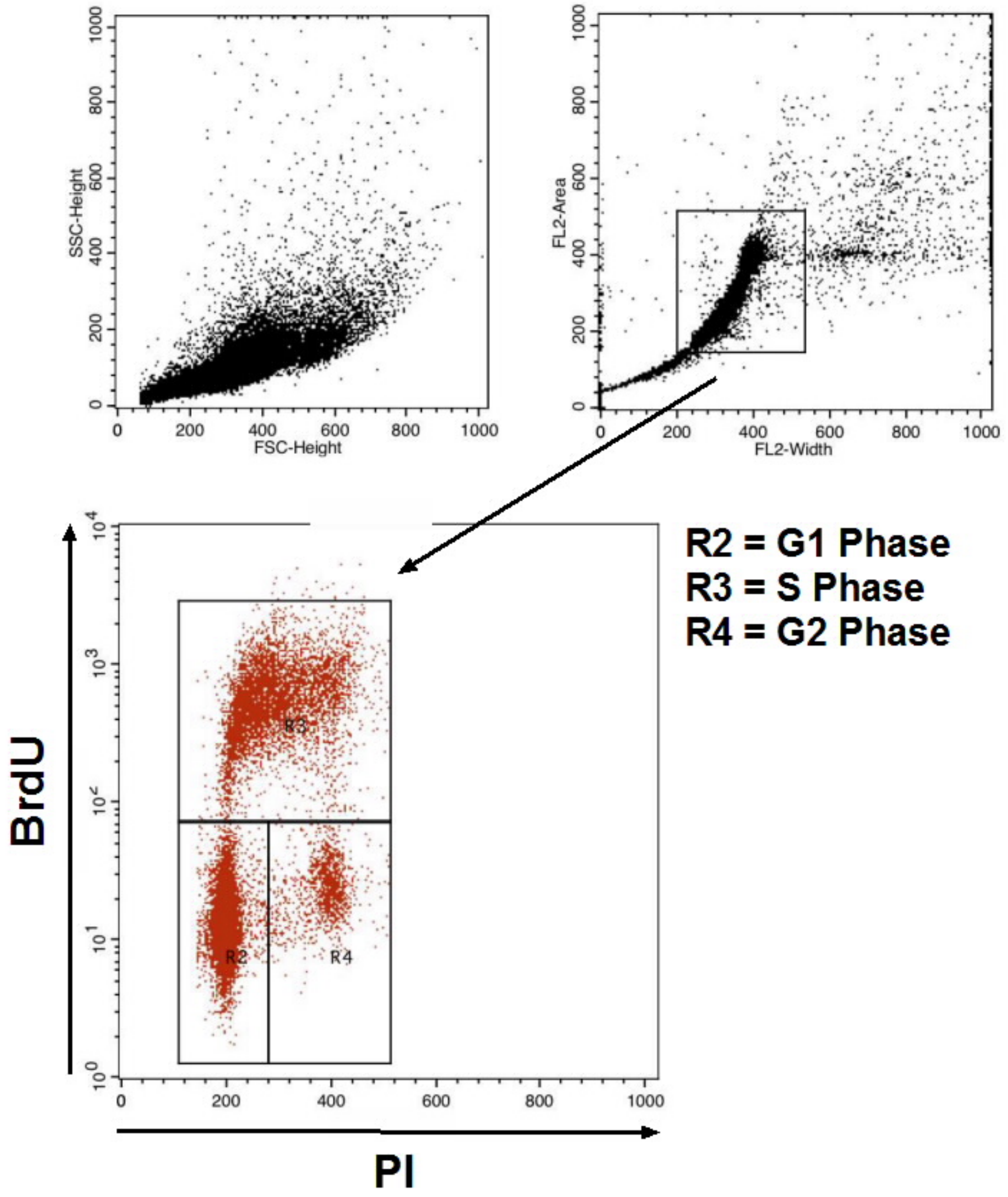

Supplementary Table 1 Core SUVARness signature genes in mice and men

Mouse and human gene symbols as well as NCBI ENTREZ IDs of the 22-gene core SUVARness signature. Genes are sorted by log fold-change, with the largest difference in descending order (only applying if two genes had the same log fold-change, secondary ordering was done by ascending *p* values).

Gene Name	Mouse Symbol	Mouse ENTREZ ID	Human Symbol	Human ENTREZ ID
FK506 binding protein 9	Fkbp9	27055	FKBP9	11328
coagulation factor II (thrombin) receptor	F2r	14062	F2R	2149
follistatin-like 1	Fstl1	14314	FSTL1	11167
phospholipid phosphatase 3	Plpp3	67916	PLPP3	8613
NCK-associated protein 1	Nckap1	50884	NCKAP1	10787
cathepsin C	Ctsc	13032	CTSC	1075
wntless WNT ligand secretion mediator	Wls	68151	WLS	79971
paired related homeobox 1	Prrx1	18933	PRRX1	5396
matrix metallopeptidase 9	Mmp9	17395	MMP9	4318
integrin alpha M	Itgam	16409	ITGAM	3684
glutathione peroxidase 8 (putative)	Gpx8	69590	GPX8	493869
sphingosine phosphate lyase 1	Sgpl1	20397	SGPL1	8879
serum/glucocorticoid regulated kinase 1	Sgk1	20393	SGK1	6446
FK506 binding protein 10	Fkbp10	14230	FKBP10	60681
growth arrest specific 1	Gas1	14451	GAS1	2619
syndecan 2	Sdc2	15529	SDC2	6383
cadherin 2	Cdh2	12558	CDH2	1000
CD274 antigen	Cd274	60533	CD274	29126
family with sequence similarity 114, member A1	Fam114a1	68303	FAM114A1	92689
sorting nexin 7	Snx7	76561	SNX7	51375
XIAP associated factor 1	Xaf1	327959	XAF1	54739
CD38 antigen	Cd38	12494	CD38	952

Supplementary Methods

List of primers

Following primers were used for detection of the Eμ-*myc* transgene and control α-tubulin.

Eμ-myc 830 bp

Eμ-myc fwd 5'-CAG CTG GCG TAA TAG CGA AGA G-3'

Eμ-myc rev 5'-CTG TGA CTG GTG AGT ACT CAA CC-3'

α-Tubulin 530 bp

α-Tubulin fwd 5'-CGC GAG TGC ATT TCA GTC C-3'

α-Tubulin rev 5'-TCC CAG TGA TAA GCT GCT CT-3

Microarray data processing

Clinical trial-like mouse lymphoma model. Raw expression data in CEL files from untreated and CTX-challenged mice were imported into R and normalized using RMA implemented in the package *oligo*. Potential inconsistencies between different measurement batches were reduced using Empirical Bayes methods (ComBat) in the *sva* R package and the CEL files scan date as batch factor. Treatment and CTX response groups were included as covariates in batch correction to preserve biological information.

Eμ-myc dataset for DLBCL subtype analysis. Expression data of 39 primary Eμ-*myc* lymphomas from our clinical-trial like model were combined with expression profiles from publicly available primary Eμ-*myc* lymphoma (GSE40760). Raw CEL files were imported separately for samples processed on different array platforms and normalized using RMA without quantile normalization. Subsequently, the different expression matrices were combined as described below and the resulting matrix quantile normalized. Lastly, batch effects were reduced using Empirical Bayes methods (ComBat) in the *sva* R package and the CEL files scan date as batch factor.

TIS. Raw expression data in CEL files from our previously published Suv39h1$^-$;bcl2 lymphomas[1] (GSE44355), control;bcl2 lymphomas and Suv39h1$^-$;bcl2 transduced with 4OHT-inducible Suv39h1 (Suv39h1:ER;bcl2) were imported and normalized using RMA implemented in the package *oligo.* Potential inconsistencies between different measurement batches were reduced using Empirical Bayes methods (ComBat) in the *sva* R package and the CEL files scan date as batch factor. Treatment and phenotype groups

were included as covariates in batch correction to preserve biological information. Phenotype groups were designated as follows: ADR-exposed control;bcl2 or ADR/4OHT-double-treated Suv39h1:ER;bcl2 lymphomas were considered senescent, while untreated and ADR-treated Suv39h1^{-};bcl2 or Suv39h1:ER;bcl2 lymphomas were considered non-senescent.

Combination of expression datasets

In order to combine expression data from different datasets, probesets were collapsed to the gene level. We followed the strategy by Monti et al.[2] and considered the correlation of probesets representing the same gene to decide whether to average probesets ($c > 0.2$) or to use the probeset with highest average expression across samples ($c \leq 0.2$). Probesets without known annotation were removed, non-human annotations humanized as described below (see "conversion of homologue genes" below) and genes present on all platforms matched.

Principal components analysis, hierarchical clustering and heatmaps

Principal components analysis (PCA) was performed using the *pcaMethods* package. Hierarchical clustering was performed using the R package *hclust* using pearson-correlation distance and complete linkage. Data was row (gene) mean-centered and scaled to unit variance. Heatmaps were produced using the package *pheatmap*.

Differential gene expression analysis

Differential gene expression analysis was performed using *limma* and Empirical Bayes statistics. In order to focus on single genes, probesets without annotations were removed and probesets collapsed to the gene level using the probeset with highest statistical difference between the groups of interest by an unpaired t-test prior to the analysis. *P* values were corrected for multiple testing using the BH method to control for false discovery rate[3].

Conversion of homologue genes

Gene symbols were converted between organisms using data from NCBI Homologene (build 68). For conversion between mouse and human, genes without a homologue were complemented by additionally searching in NCBI using the uppercase symbol (mouse-to-human) or capitalized lowercase symbol (human-to-mouse). Genes were converted between the different identifiers using Bioconductor annotation data packages.

Stratification by average signature gene expression

Expression data were filtered for the genes of interest, row (gene) mean-centered and scaled to unit variance. Subsequently, probesets were collapsed to the gene level using the correlation-based approach as described above followed by computation of the average gene expression value for each sample and signature. Lastly, the samples were stratified based on median expression of this new meta signature expression value.

Linear predictor score (LPS) classification

The LPS classifier was implemented as presented by the Staudt lab[4]. The training and test data were combined as described above and the expression values of the test data shifted and scaled to match the mean and standard deviation of the training set. Probeset collapsing was done in the training data by using the maximum statistical significance between the sample groups and in the test data using the correlation-based approach.

For the COO subtype classification, the GSE10846 training data (CHOP-treated patients) were used. For CCC subtype classification we used the data from Monti et al.[2] and an iterative three-group variation of the LPS classifier. The data was further separated into 2/3 training and 1/3 validation data. We determined the lowest overall error rate comparing first HR *vs.* REST followed by OxPhos *vs.* BCR based on the DLBCL training and validation data. This approach was then applied to the Eμ-*myc* cohort.

Gene set enrichment analysis

GSEA[5] was performed using the R package *clusterProfiler*. Probesets were collapsed to the gene level using the correlation-based approach as described above, and unknown probesets were removed. The signal-to-noise ratio $(\mu_A - \mu_B)/(\sigma_A + \sigma_B)$ (μ = mean,

σ = standard deviation) was used as a ranking metric, and statistics were based on gene set permutations. FDR *q* values ≤ 0.05 were considered significant.

Supplementary References

1. Dorr JR*, et al.* Synthetic lethal metabolic targeting of cellular senescence in cancer therapy. *Nature* **501,** 421-425 (2013).

2. Monti S*, et al.* Molecular profiling of diffuse large B-cell lymphoma identifies robust subtypes including one characterized by host inflammatory response. *Blood* **105**, 1851-1861 (2005).

3. Hochberg Y, Benjamini Y. More powerful procedures for multiple significance testing. *Stat Med* **9**, 811-818 (1990).

4. Wright G, Tan B, Rosenwald A, Hurt EH, Wiestner A, Staudt LM. A gene expression-based method to diagnose clinically distinct subgroups of diffuse large B cell lymphoma. *Proc Natl Acad Sci U S A* **100**, 9991-9996 (2003).

5. Subramanian A*, et al.* Gene set enrichment analysis: a knowledge-based approach for interpreting genome-wide expression profiles. *Proc Natl Acad Sci U S A* **102**, 15545-15550 (2005).
