# Supplementary material for: H3K9me3-mediated epigenetic regulation of senescence in mice predicts outcome of lymphoma patients
Source: Nat Commun. 2020 Jul 20;11:3651. doi: 10.1038/s41467-020-17467-z (PMC7371731; doi:10.1038/s41467-020-17467-z)
Supplement: Supplementary file 3 — Reporting summary [file 41467_2020_17467_MOESM3_ESM.pdf]

nature research

Corresponding author(s): Clemens A. Schmitt
Last updated by author(s): June 03, 2020

Reporting Summary

Nature Research wishes to improve the reproducibility of the work that we publish. This form provides structure for consistency and transparency in reporting. For further information on Nature Research policies, see our Editorial Policies and the Editorial Policy Checklist.

Statistics

For all statistical analyses, confirm that the following items are present in the figure legend, table legend, main text, or Methods section.

n/a	Confirmed	
☐	☒	The exact sample size (n) for each experimental group/condition, given as a discrete number and unit of measurement
☐	☒	A statement on whether measurements were taken from distinct samples or whether the same sample was measured repeatedly
☐	☒	The statistical test(s) used AND whether they are one- or two-sided<br>*Only common tests should be described solely by name; describe more complex techniques in the Methods section.*
☐	☒	A description of all covariates tested
☐	☒	A description of any assumptions or corrections, such as tests of normality and adjustment for multiple comparisons
☐	☒	A full description of the statistical parameters including central tendency (e.g. means) or other basic estimates (e.g. regression coefficient) AND variation (e.g. standard deviation) or associated estimates of uncertainty (e.g. confidence intervals)
☐	☒	For null hypothesis testing, the test statistic (e.g. F, t, r) with confidence intervals, effect sizes, degrees of freedom and P value noted<br>*Give P values as exact values whenever suitable.*
☒	☐	For Bayesian analysis, information on the choice of priors and Markov chain Monte Carlo settings
☒	☐	For hierarchical and complex designs, identification of the appropriate level for tests and full reporting of outcomes
☒	☐	Estimates of effect sizes (e.g. Cohen's d, Pearson's r), indicating how they were calculated

Our web collection on statistics for biologists contains articles on many of the points above.

Software and code

Policy information about availability of computer code

Data collection	Based on previous experience with the Eμ-myc transgenic mouse lymphoma model, sample sizes typically reflect three to five, in some experiments also much higher numbers of individual primary tumors as biological replicates. For assessing long-term outcome after in vivo-treatments, seven or more tumor-bearing animals per arm were used. All quantifications from staining reactions were carried out by an independent and blinded second examiner and reflect at least three samples with at least 100 events counted (typically in three different areas) each, and a t-test was applied.
Data analysis	Survival analysis was done using the survival package in R. Statistical significance of differences in the survival times were assessed using the log-rank test. Unless otherwise stated, a P value < 0.05 was considered statistically significant. For multiple testing corrections the method by Benjamini & Hochberg (BH) to control for false discovery rate was applied63. Bioinformatics Analysis was performed in R 3.5.0 & Bioconductor 3.7 using various R packages. In order to ensure reproducible results, the R workspace was initiated with a random seed of 1.

For manuscripts utilizing custom algorithms or software that are central to the research but not yet described in published literature, software must be made available to editors and reviewers. We strongly encourage code deposition in a community repository (e.g. GitHub). See the Nature Research guidelines for submitting code & software for further information.

Data

Policy information about availability of data

All manuscripts must include a data availability statement. This statement should provide the following information, where applicable:

- Accession codes, unique identifiers, or web links for publicly available datasets
- A list of figures that have associated raw data
- A description of any restrictions on data availability

The mouse model-derived raw microarray data – from our previously published control;bcl2, Suv39h1-;bcl2 and Suv39h1-;bcl2 transduced with 4OHT-inducible

Suv39h1 (Suv39h1:ER;bcl2) lymphomas – were deposited at the Gene Expression Omnibus (GEO) repository of the National Center for Biotechnology Information under accession number GSE134753. Data from our clinical-trial like model were deposited under accession number GSE134751. Expression data of 39 primary Eμ-myc lymphomas from our clinical trial-like model were combined with expression profiles from publicly available primary Eμ-myc lymphoma (GSE40760). Expression data of DLBCL patients are publicly available from NCBI GEO, comprising GSE10846, GSE4475, GSE4732 and GSE31312. In addition, gene expression profiles of DLBCL patients from the Shipp lab for CCC DLBCL distinction were obtained from https://portals.broadinstitute.org/cgi-bin/cancer/publications/pub_paper.cgi?mode=view&paper_id=102 (Ref. 53) and for GSE98588 from NCBI GEO. Raw CEL files were downloaded and processed using RMA implemented in the R package oligo and batch effects between scan dates reduced using ComBat in the sva package.

Field-specific reporting

Please select the one below that is the best fit for your research. If you are not sure, read the appropriate sections before making your selection.

[x] Life sciences [ ] Behavioural & social sciences [ ] Ecological, evolutionary & environmental sciences

For a reference copy of the document with all sections, see nature.com/documents/nr-reporting-summary-flat.pdf

Life sciences study design

All studies must disclose on these points even when the disclosure is negative.

Sample size

Based on previous experience with the Eμ-myc transgenic mouse lymphoma model (References 27, 28, 30, 33 - 38 in the manuscript), sample sizes typically reflect three to five, in some experiments also much higher numbers of individual primary tumors as biological replicates. For assessing long-term outcome after in vivo-treatments, seven or more tumor-bearing animals per arm were used. Sample size for each figure panel is mentioned in the corresponding figure legend.

Data exclusions

No data was excluded.

Replication

Individual lymphomas were propagated in up to two strain-matched, non-transgenic (i.e. genetically non-engineered wild-type), fully immuno-competent 6-8-week-old mice each via tail vein injection of 106 viable cells (notably, in rare cases, discordant NR/RP responses of the two same-lymphoma recipients may produce unequal medians of the stratified response groups). For in vitro experiments, sample sized and number of technical replicates are mentioned in corresponding figure legend. Most of replications were successful and the variations of assay results are described in the text, as well as in the form of statistical parameters such as standard deviation.

Randomization

Murine lymphoma samples were allocated into never-relapse (NR) or relapse-prone (RP) groups according to the treatment result after a single intraperitoneal dose of cyclophosphamide (300 mg/kg body weight) or CHOP (i.e. 150 mg/kg CTX, 3.3 mg/kg ADR, 0.5 mg/kg Vincristine, and 200 μg/kg Prednisone) at the time well-palpable LN enlargements (i.e. about 8-10 mm in diameter) had formed. Treatment responses were monitored during regular visits at least twice a week, for a maximum of a 100-day observation period. Examination of mice included a general inspection and palpation of the typically affected LN regions (i.e. cervical, pre-scapular/axillar and inguinal) as time-to-relapse (TTR), i.e. progression-free survival (PFS), or reflect the time between treatment and unexpected death of the animal or a preterminal disease stage, collectively measured as overall survival (OS).

Blinding

Researchers were blinded to group allocation during data collection in all experiments in which the subjective view could influence the results. All quantifications from staining reactions were carried out by an independent and blinded second examiner and reflect at least three samples with at least 100 events counted (typically in three different areas) each, and a t-test was applied.

Reporting for specific materials, systems and methods

We require information from authors about some types of materials, experimental systems and methods used in many studies. Here, indicate whether each material, system or method listed is relevant to your study. If you are not sure if a list item applies to your research, read the appropriate section before selecting a response.

Materials & experimental systems

n/a	Involved in the study
[ ]	[x] Antibodies
[x]	[ ] Eukaryotic cell lines
[x]	[ ] Palaeontology and archaeology
[ ]	[x] Animals and other organisms
[x]	[ ] Human research participants
[x]	[ ] Clinical data
[x]	[ ] Dual use research of concern

Methods

n/a	Involved in the study
[x]	[ ] ChIP-seq
[ ]	[x] Flow cytometry
[x]	[ ] MRI-based neuroimaging

Antibodies

Antibodies used

Primary antibodies against gamma-H2AX (Cell Signaling Technology [CST], clone 20E3, #9718), p53-P-Ser18 (CST, #9284S), p53 (Leica Biosystems, #NCL-p53-CM5p), p16INK4a (Santa Cruz Biotechnology, Fclone F-12, #sc-1661), H3K9me3 (Abcam, #ab8898), LSD1 (CST, clone C69G12, #2184), JMJD2C (Abcam, #ab85454) and a-Tubulin (Sigma, clone B-5-1-2, #T5168). Anti-mouse or anti-rabbit horseradish peroxidase-conjugated antibodies were used as secondary antibodies (GE Healthcare, #RPN4301 and #NA931V,

respectively). Antigen detection by immunohistochemistry was performed with antibodies against Ki67 (Dako, clone MIB-1, TEC3) and H3K9me3 (Abcam, #ab8898).

Validation

gamma-H2AX (CST #9718): reactivity to H (human), M (mouse), R (rat), Mk (monkey). Application for WB (western blot), IHC (immunohistochemistry), IF (immunofluorescence), F (flow cytometry). Certificate of analysis https://media.cellsignal.com/coa/9718/17/9718-lot-17-coa.pdf

p53-P-Ser18 (CST #9284): reactivity to H, M, R, Mk. Application for WB, IP (immunoprecipiataion), ChIP (chromatin IP). Certificate of analysis https://media.cellsignal.com/coa/9284/21/9284-lot-21-coa.pdf

p53 (Leica Biosystems, #NCL-p53-CM5p): reactivity to M. Application for WB, IHC, HIER. https://shop.leicabiosystems.com/global_en_US/ihc-ish/ihc-primary-antibodies/pid-p53-protein-cm5

p16INK4a (Santa Cruz Biotechnology, #sc-1661): reactivity to H, M, R. Application WB, IP, IF, IHC, ELISA. Validation info at https://datasheets.scbt.com/sc-1661.pdf

H3K9me3 (Abcam, #ab8898): reactivity to H, M, R, Chicken, Saccharomyces cerevisiae, Xenopus laevis, Drosophila melanogaster, Indian muntjac, Xenopus tropicalis, Cyanidioschyzon merolae. Application for IHC, ICC/IF, ChIP, WB, ChIP/Chip, F, CHIPseq. Validation info at https://www.abcam.com/histone-h3-tri-methyl-k9-antibody-chip-grade-ab8898.html

LSD1 (CST, #2184): reactivity to H, M, R, Mk. Application for WB, IHC, IF, IP, ChIP. This antibody has been validated using SimpleChIP® Enzymatic Chromatin IP Kits. 45 product citations available https://en.cellsignal.de/products/primary-antibodies/lsd1-c69g12-rabbit-mab/2184

JMJD2C (Abcam, #ab85454): reactivity to H, M. Application for WB, IHC. Validation info at https://www.abcam.com/kdm4c-gasc1-jmjd2c-antibody-ab85454.html

a-Tubulin (Sigma, #T5168): reactivity to H, M, R, Mk, chicken, kangaroo rat, sea urchin, Chlamydomonas, bovine. Application for WB, IF. Validation info at https://www.sigmaaldrich.com/catalog/product/sigma/t5168?lang=de&region=DE&gclid=EAIaIQobChMItbHmjKGu6QIVArTtCh0nUQgHEAAYASAAEgKndfD_BwE

Ki67 (Dako, TEC3): reactivity to H, M. Application for WB, IHC. Validation info at https://www.chem.agilent.com/cs/library/packageinsert/public/SSM7240CEEFG_02.pdf

Animals and other organisms

Policy information about studies involving animals; ARRIVE guidelines recommended for reporting animal research

Laboratory animals	Mus musculus, strain C57BL/6. As transplantation recipients, 6 - 8 weeks old female mice were used. Primary lymphomas from genetically modified animals of mixed gender were prepared according to the course of the disease and regulations. Mice were maintained under a 12 h light/12 h dark cycle in specific pathogen-free (SPF) conditions in IVC cages at 22-24 °C and 50 - 60% humidity. All mice were fed normal chow diets and water ad libitum.
Wild animals	This study did not involve wild animals.
Field-collected samples	This study did not involve field-collected samples.
Ethics oversight	Animal experiments were approved by the animal experiment committee (Tierversuchskommission) of the local governmental review office LAGeSo (Landesamt fur Gesundheit und Soziales) Berlin and performed according to EU and national institutional regulations.

Note that full information on the approval of the study protocol must also be provided in the manuscript.
